# RNA Aptamer Evolution: Two Decades of SELEction

**DOI:** 10.3390/ijms12129155

**Published:** 2011-12-08

**Authors:** Guillermo Aquino-Jarquin, Julia D. Toscano-Garibay

**Affiliations:** 1 Unit of Research on Oncological Disease, Children’s Hospital of Mexico Federico Gomez, Mexico City 06720, Mexico; E-Mail: guillaqui@himfg.edu.mx; 2 Regenerative Medicine Laboratory. Research Direction, Mexico’s Juarez Hospital, Mexico City 07760, Mexico

**Keywords:** non-coding RNAs, aptamers, SELEX, *in vitro* selection, *in vitro* evolution

## Abstract

Aptamers are small non-coding RNAs capable of recognizing, with high specificity and affinity, a wide variety of molecules in a manner that resembles antibodies. This class of nucleic acids is the resulting product of applying a well-established screening method known as SELEX. First developed in 1990, the SELEX process has become a powerful tool to select structured oligonucleotides for the recognition of targets, starting with small molecules, going through protein complexes until whole cells. SELEX has also evolved along with new technologies positioning itself as an alternative in the design of a new class of therapeutic agents in modern molecular medicine. This review is an historical follow-up of SELEX method over the two decades since its first appearance.

## 1. Long Story Short, the Birth of Selection Methods

In 1990, a mutation experiment was conducted by Tuerk and Gold [1] to explain the nature of the translational regulation exerted by the phage T4 replicase over its own messenger. They randomized a stretch of eight nucleotides within the regulatory loop of the mRNA and systematically exposed the resulting pool of sequences to the replicase. Two hairpins out of 4^8^ (~65,536) possible combinations were isolated that bind with very similar affinity. This experiment defined the SELEX method for the first time and allowed us to envisage nucleic acids as flexible ligands potentially useful in protein recognition.

In parallel, Ellington and Szostak [2] utilized the same strategy while seeking a way to explain the existence of active sites. They wondered whether RNA molecules had the ability, like proteins, to form stable surfaces that provided “pockets” for specific interaction with small molecules (e.g., organic dyes) and designated the resultant ligands as APTAMERS, a term derived fromthe combination of the Latin word “*aptus*” (meaning to fit) and the Greek word *mers* (particle).

Although SELEX was not meant to be a method for the screening of oligonucleotides with novel functions, it rapidly was visualized and adapted for this purpose. The basic SELEX method has been *evolved* to achieve a number of specific objectives [3,4]. In general, it seems to be a progress in which, after grounds were settled, the selection libraries started to be modified in order to improve their resistance *in vitro*. Afterwards, once sufficient functionality and resistance were proved, the aptamers in the cell interior were tested. In these experiments, aptamers were shown to be suitable for cell conditions and the major concern was how to regulate and detect them inside the cell. Finally, after 13 years, aptamers were ready to be used as biotechnological tools and the view was focused on improving the method to make it more efficient, incorporating new technologies. In this review we provide a historical overview of most of the SELEX variants using the first published reports as references (Table 1). It is important to mention that some of these variants of SELEX were developed to obtain DNA aptamers but the same methods can be applied for non-coding RNA libraries and are were described as part of the evolution of SELEX.

## 2. Setting the Ground for First Selections (1990–1994)

### 2.1. Classic SELEX

The data derived from the pioneering works mentioned above, led to the generalization of SELEX as a useful method for the identification single-stranded oligonucleotides folding into structures that could interact with different molecules. Then, the SELEX process was described as an *in vitro* evolution of nucleic acid molecules until have with high specificity to target molecules.

**Figure 1:**
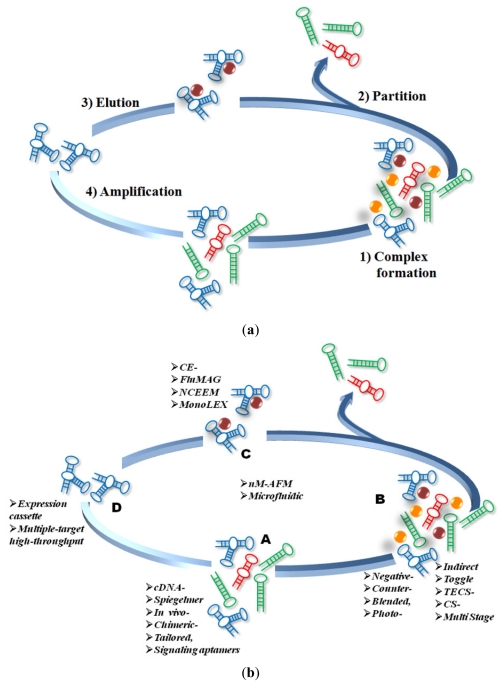
SELEX evolution. This scheme shows the basic steps of SELEX (**a**) and the main modifications done over two decades (**b**). The methods indicated on (**b**) are positioned on each aspect of SELEX where modifications were proposed. The techniques on the center of (**b**) represent major changes—at least in three aspects—of classic SELEX. A: Library design, B: Target type, C: Partition and D: Elution and amplification.

The classic SELEX method involved steps of iterative binding, partitioning and amplification applied to a mixture of candidate oligonucleotides through a general scheme of four phases until virtually any desired criteria of affinity and selectivity could be achieved. The initial pool of nucleic acids, was preferably designed with a randomized segment in the middle section of its sequence [1]. In the first phase, specific complexes are formed by incubation of the pool with the target under controlled binding conditions. The second phase, and probably the most important, is the partitioning of unbound nucleic acids from the mixture. The third phase involves the dissociation of the nucleic acid-target complexes, and finally, the last phase comprehends the amplification of successful nucleic acids to yield an enriched group of aptamers. This description corresponds to what was named a selection cycle, in this way, by reiterating the steps of binding, partitioning, dissociating and amplifying through as many cycles as desired, it could be possible to yield highly specific and affinity aptamers to the target molecule (Figure 1a). A typical aptamer is 5–15 kDa in size (15–45 nucleotides), binds its target with nanomolar to sub-nanomolar affinity and can discriminate among closely related targets. Interesting, a series of structural studies have shown that aptamers are capable of using the same types of binding interactions (e.g., hydrogen bonding, electrostatic complementarities, hydrophobic contacts, steric impediments) that drive affinity and specificity in antibody-antigen complexes. Despite the fact that nucleic acids are formed by only four nucleotides, has proved that it is enough to acquire a variety of bi- and three-dimensional structures and is sufficient chemical versatility to be compared with proteins, forming specific binding pairs with virtually any chemical compound.

Since this first description, more or less twenty years ago, more than 25 variants of SELEX process have been described that modified the basic steps of the original selection procedure, each on specific aspects (Figure 1b).

### 2.2. Negative-SELEX

Even though SELEX raised expectation as a promising screening method, during the first two years after *classic* procedure, other selections experiments resulted in populations of oligonucleotides without exclusive affinity. Isolated ligands recognized components unavoidably present in the selective environment. This behavior led to a modification of the method to eliminate unspecific interactions, which was named negative-SELEX. The process excluded those aptamers adsorbed by the matrixes used for immobilization of selection targets with the purpose of enriching the population with sequences that could form complexes only with the target itself. In this adjustment of classic-SELEX, the screening pools are loaded onto the matrix alone and after an incubation period the flow-through is mixed with the immobilized target to isolate specific aptamers. Negative-SELEX was first put forward by Ellington and Szostak in 1992 [5]. They used it after several selection cycles to eliminate sequences binding to the agarose matrix employed as purification support. Compared to SELEX without negative selection, the affinity of the resultant aptamers was about 10 times higher. This outcome testified the feasibility of negative-SELEX in practical application, especially for the screening process with immobilized targets.

### 2.3. Counter-SELEX

Once non-specific interactions with the system of selection were eliminated, changes were made to the procedure to increase the capacity of aptamers to discriminate between structurally similar molecules. Counter-SELEX improves aptamers selectivity by excluding RNA molecules with co-affinity for molecules that are similar to the targets. The idiographic processes are similar to the negative SELEX; simply exchange the matrixes with the analogues of the target. This method was established by Jenison *et al.* when identifying RNA aptamers for bronchodilator theophylline [6]. They selected aptamers for caffeine, using theophylline—which differs only by a methyl group at nitrogen atom N7—as the counter-target, and finally aptamers were obtained that could bind only to theophylline. Counter-SELEX had the same purpose as negative-SELEX, which is to eliminate aptamers recognizing other targets, but in this case by using analogue targets it was also possible to increase the selectivity of the obtained sequences [6,7].

## 3. Improving the Libraries (1995–1996)

### 3.1. Blended SELEX

Afterwards, libraries began to be modified thinking ahead on providing the necessary resistance for *in vivo* applications. Blended SELEX appeared as a method which leads the aptamers towards a specific region of the target, through fusing them to no-nucleic acid counterparts. This method was established by Smith *et al.* in 1995 [8], as an alternative to enrich the combinatorial pool for drug discovery. They connected an inhibitor of the neutrophil elastase (valP) with a 5′-linker DNA or splint-oligonucleotide, which can hybridize with the 3′end of the randomized RNA pool. Therefore, this inhibitor could be joined to the random sequence to form a “blended pool” suitable for SELEX. In their experiment, blended molecules specifically attached to elastase through valP portion, dragging the possible aptamers into the interaction and promoting the formation of a new covalent bond between valP and the active site of the enzyme. The RNA-DNA-valP-elastase complex was separated by PAGE and the RNA molecules were recycled for the following process of SELEX [8]. Incorporation of non-nucleic acid functional units to produce blended SELEX pools increases the repertoire of structures and interactions available to produce high affinity binding ligands. Various types of units can be incorporated to produce a spectrum of molecular and functional structures.

### 3.2. Photo-SELEX

Simultaneously, another alternative for the modification of libraries that allowed an increase in the affinity and stability of aptamer-target complexes, was first reported by Jensen *et al.* [9], who obtained aptamers for Rev Protein of HIV-1. Photo-SELEX uses photosensitive nucleotides to promote covalent cross-link with other molecules under a certain wavelength light. 5-IU or 5-BrdU was mixed into the screening pool, then the modified library was incubated with the target, the mixture was exposed to ultraviolet radiation to make the cross-link reaction happen. Then, the mixture could be separated by PAGE. The wavelength of the ultraviolet radiation and the irradiation time should be optimized to ensure that only specific sequences could react with the targets. The incorporation of a photoreactive chromophore (5-IU or 5-BrU) into a randomized RNA library allowed the *in vitro* selection of ligands which not only could bind with high affinity but retained specificity for its target.

### 3.3. cDNA-SELEX

In the same year (1995), another library modification strategy was proposed by Dobbelstein *et al.* [10] using sequences from a natural source like organisms genomes, unlike previous techniques where synthetic pools were employed to search for specific interactions. This method was called cDNA-SELEX and consists on the use of total cell RNA from Human B-cell lymphoma as the screening pool and L22 protein as the target, searching for discriminating sequences. Initially an immunoprecipitation of total RNA-L22 complexes with a specific antibody was performed, followed by SELEX amplification for binding sequences present in the immunoprecipitate. This analysis revealed three sites on 28S ribosomal RNA that have the potential to interact with L22, one site on 18S ribosomal RNA, and two RNA segments that are not in current sequence databases. This method provided a new strategy to study the regulation networks between protein and nuclear acid in a manner similar to proteomic procedures that would be developed years later.

### 3.4. Spiegelmer Technology

When it was first projected that aptamers could be used in medical treatment, it was a key point to improve their stability *in vivo* because the half-lives of DNA and RNA are all short *in vivo*. Typically, the principal strategy to increase nuclease resistance was, until that moment, to modify the backbone of nucleic acids using chemical modification (phosphorothioates, methylphosponates or phosphoroamidates) [11]. However, in 1996 [12] another method was created to confer stability to aptamers designated as Spiegelmer Technology. With the help of the optical activity of chiral molecules, Spiegelmer Technology screens aptamers from dextrorotatory oligonucleotide pool. Then, the corresponding levorotatory oligonucleotide can recognize the target molecule and could not be cleavage by nucleases, which make them attractive molecules for *in vivo* application. The aptamers obtained via this strategy are also called Spiegelmers. However, this method only suits the screening for small molecules with optical activity.

## 4. First Attempts to Enter the Cell Environment (1997–1999)

### 4.1. In Vivo SELEX

After almost a decade of development, SELEX processes reached a point where aptamers were readily selected and started to diversify towards the use of complex selection environments looking for new functionalities, other than recognizing. At that time, the goal was to transfer *in vitro* selection into a cellular context. In 1997 the first report of a selection made inside mammalian cells was done by Coulter *et al*. [13]. This approach is modeled on the *in vitro* SELEX procedure and uses transient transfection and an iterative procedure to enrich RNA-processing signals in culture. The authors incorporated a randomized sequence flanked by a duplication of an intronic region that is naturally exscinded from the pre-mRNA of a minigene (SXN13). Then, the construct was transfected in a transient fashion into QT35 cells to utilize the endogenous splicing machinery. The insertion of this library generated potential splicing sites that allowed the recovery of two final transcripts: those with and without a new exon constituted by the randomized sequence (R). Total mRNA was isolated and a RT-PCR reaction was performed to amplify the “R” segment that was cloned into the same initial minigene construct to complete the equivalent steps of an *in vitro* selection cycle [13]. With this approach, after the third selection cycles it was possible to find splincing enhancers sites unknown at that time.

### 4.2. Chimeric SELEX

Once SELEX process was transferred within the cell, the search for aptamers with complex functions began. Burke and Willis [14] performed, in 1998, experiments to fuse a series of aptamers previously selected every one able to recognize an independent target (Cloramphenicol, adenosine or Co-A). They used 2–3 aptamers for each target, creating a “mini-library” of 22 combinations. Their findings proved that the newly formed molecules or chimeras (hence the name chimeric-SELEX) retained a reduced recognition activity for both targets, probably due to misfolding. However, applying a dual selection pressure to the recombined populations, best suited combinations were produced that bind to both targets [14]. In this sense, the chimeric molecules are useful to improve affinities for a target molecule, to enhance assembly of bifunctional molecules and to recapitulate *in vitro* a possible evolutionary mechanism that might happening in the hypothetic RNA world. Also, chimeric-SELEX had served as a precedent for selections directed to promote reactions between two adjacent molecules.

### 4.3. Multi Stage SELEX

A variant of chimeric SELEX was developed by Wu *et al.* in 1999 [15] by iterating the strategy previously described. The approach results in a multi-stage SELEX of five steps. Stage one starts on the selection of parental aptamers from randomized libraries. Then, in stage two, a counter-selection step is used to avoid cross-reaction between each selection target (cibacron blue and cholic acid). In stage three, obtained aptamers are fused to each other (named allosteric DNAs) and reselected to isolate the most affinity allosteric pairs. Stage four was to separate binding regions and returned to the counter-selection like stage two. In stage five, new allosteric-DNA combinations are re-joined and re-selected to later be cloned and characterized. The isolated aptamers could bind either cibacron blue or cholic acid fixed in columns and eluted by any of the free targets (either cibacron or cholate). The novelty of multi stage SELEX relies on the fact that only two steps of variability (bi-aptamer mini-library construction) are introduced to create molecules with two domains which activity depends on interaction with the counter-target.

## 5. Aptamer Regulation and Detection (2000–2003)

### 5.1. Signaling Aptamers

After experimenting with different library designs and verifying for intra-cellular functionality, the possibility of evaluate the potential applications aptamers *in vivo* and *in vitro* became tangible. Some of the selection processes started to use the acquire knowledge on aptamer modification to explore its application as biosensor, through methods that coupled their molecular recognition abilities with signal transduction. Signaling aptamers were initially obtained by Jhaveri *et al.* in 2000 when they inserted limited number fluoresceinated uridines into a library selected to identify ATP. The obtained aptamers were able to detect its target with high sensitivity (25 μM) and with as little as one modified uridine [16]. Later, methods were based on molecular beacon technology, that is, on the conformational change occurring when two oligonucleotides hybridize with each other causing two fluorescent reporters to separate [17]. For aptamers, the conformational change is produced by interaction with the selection target, ultimately causing a loss of fluorescence or a dequenching of a fluorophore. The basic design of a SELEX for *aptamer beacons* starts with a library that had been amplified using a 5′-labeled primer (e.g., fluorescein), and then hybridized with a capture oligonucleotide that is biotynilated on the 3′-end and coupled to a particular quencher in the 5′-end. Hybrids are recovered using streptavidin beads and mixed with the target, in such way that only those sequences forming specific interactions are released and dequenched. When the selection procedure is over, each aptamer has the property of emitting a signal proportional to target concentration.

### 5.2. Indirect SELEX

The ability to modulate the aptamer function *in vivo* was another desirable characteristic for its application. In this regard, a selection was developed by Kawakami [18] in 2000 using a divalent cation (Zn^2+^) as inductor to form new structural motifs that can recognize naturally RNA-interacting proteins (Tat of HIV-1) and evade the isolation of consensus regions. Authors collected RNAs that bound to Tat by using a nitrocellulose filter. Sequences of the chosen RNAs were determined after 6 and 12 rounds of selection. After SELEX, many unique sequences were isolated from the library in presence of Zn^2+^ and the RNA with the most abundant sequence (e.g., clone 31) recognized Tat protein tightly only when Zn^2+^ was present in the mixture. From the data of secondary structure determination, it was possible to say that Zn^2+^ responsive Tat-aptamer should require a relatively large region of the sequence to establish a tertiary interaction of several motifs in order to perform the binding [18].

### 5.3. Toggle SELEX

On the other hand, while libraries became more intricate, targets used for selection gradually were increasing in size, but the consequence was that the affinity, in many cases, fell; probably because SELEX directed against large molecular surfaces resulted in the isolation of multiple sequences that attached to various sites (or epitopes), some with higher affinity than others. Therefore, when individual sequences were isolated, the chances of have found a weak-interacting aptamer were bigger. After this observation, SELEX started to be modified so each aptamers recognizing an epitope had the highest interaction possible. Toggle-SELEX was proposed in 2001 to yield a number of aptamers acting as “polyclonal” populations that eventually could be separated into stronger individual aptamers. Two studies under this strategy appeared that year, Bianchini *et al.* [19] selected several RNA aptamers for ERK2 using initially a small peptide (comprising two phosphorylation sites) as an intermediate target that was fixed on a nitrocellulose membrane. They tested this sub-population for positive interaction and then the target was switched for ERK2 complete protein extracted from T84 cell cultures. As the authors stated, by using a crude extract they ensured selection on the basis of specificity rather than affinity, with the important consequence that the latter increased considerably (63 nM). Only two steps were made to obtain a very specific mixture of sequences and individual aptamers, also showing that selection methods could begin to be simplified.

The second experiment was performed by White *et al.* [20], who described a selection in which RNA aptamers were obtained to recognize both human and porcine thrombin. Initial library was incubated with a mix of both proteins to enrich the population with thrombin-recognizing aptamers. This “pre-selected” library was then incubated with one protein in an alternately way, until sequences joining common region on both species were isolated. In parallel, two selections directed to either human or porcine protein were conducted as comparative populations. At the end, a family of RNA aptamers was obtained from toggle-SELEX that had cross-reactivity and a high affinity analogous to aptamers isolated form targets used individually. This procedure was then proposed as a cheaper method to obtain molecules suitable for animal testing.

### 5.4. Expression Cassettes

The next consideration taken into the path towards aptamer application was the design of appropriate delivery methods. One approach was published in 2002 by Martell *et al.* [21] where a previously isolated aptamer to the E2F1 transcription factor was coupled to a Pol III promoter as an expression unit (or cassette) in a plasmidic DNA. The construct contained a promoter, a tRNA sequence and the aptamer flanked by randomized regions. When the transcript was generated, tRNA structure stabilized the aptamer and the randomized stretches formed a stem flexible enough to allow the formation of the proper configuration for target recognition. This expression cassette yielded RNAs that bind E2F with high affinity (IC50 of 15 nM) without sacrificing its structure and which can be stably expressed at high levels in mammalian cells [21]. The advantages of this SELEX included protection of the aptamer and high levels of expression and functionality at the cell interior, constituting the first step in the design of aptamer for therapeutic purposes.

### 5.5. Tailored SELEX

Artificial synthesis was another concern on the quest for application because larger sequences were more expensive when produced on a large scale. Also, if fixed regions were involved in the recognition of the target, trying to truncate an aptamer in a post-SELEX manner might have represented a problem. In addition, modifications used to add resistance, such as mirror-images (Spiegelmer), incremented costs dramatically and large oligonucleotides were less efficiently synthesized. To overcome these issue a method, named Tailored-SELEX, that permitted the isolation of short ligands was developed by Vater *et al.* eight years ago [22], it was based on the design of cleavable primer-hybridization sites allowing to elimination of fixed sequences after the amplification of the library. The design resulted in aptamers with a randomized region flanked only for 10-known nucleotides. This approach was validated by identifying an inhibiting Spiegelmer that acted on a migraine relate peptide (α-CGRP) with an IC50 of 3 nM. Conveniently, Tailored-SELEX provided a way to obtain short sequences that can be tested more rapidly in biological systems [22].

## 6. Updating *SELEX* Method with Modern Technologies (2004–2011)

### 6.1. CE-SELEX

At his point, aptamers appear to be easily obtained and had passed the test for surviving cellular-conditions, joining very specifically to their targets. But although SELEX was being perceived as a flexible method it was yet almost artisanal. In this manner, a new episode on aptamers history started with the coupling of new devices and technologies into the general scheme. The goal of the next modifications was then to make SELEX a more standardized and effective screening method. Capillary Electrophoresis selection (CE-SELEX) is one method that used for the first time sophisticated equipment for the separation of aptamer-target complexes. Mendonsa *et al*. [23] isolated DNA aptamers recognizing human IgE by means of differential electrophoretic migration due to the size dissimilarity between the free and complexed populations. After each CE-partitioning step, recovered sequences were amplified and ssDNA separated prior to the next incubation with the target which was the beginning a new selective round. The progress of SELEX was measured by determining the dissociation constant of final populations; very specific aptamers were obtained (29 nM) and standard deviation (6 nM) shows a very narrow range of affinities. The efficiency CE leads to a high rate of enrichment, allowing isolated sequences to be obtained in only four rounds of selection. This method also decreased the number of cycles shortening selection procedure from weeks to several days.

### 6.2. FluMAG SELEX

In the matter of complex separation and affinity monitoring, Stoltenburg *et al.* developed other option in 2005 [24] that used fluorescent labeling and an target immobilized over magnetic beads. Immobilization enables malleability, smaller concentration of target, rapid and efficient separation of bound and unbound molecules, and rigorous washing steps [24]. Authors named this modified process FluMAG-SELEX, providing at the same time a methodological background for selections with targets having diverse properties and sizes, as long as they can be fixed into the beads. Initially the selection was made to isolate aptamers against streptavid in that coated magnetic beads. Complexes were separated using a magnet and aptamers amplified using a primer with a fluorescent tag. This led to a cleaner selection since unspecific interactions were easily eliminated. The advantages of the aptamers derived from FluMAG-SELEX are the possibility of applying them as biosensors useful in clinical approaches and to avoid the use of radioactive isotope-labeled libraries.

### 6.3. TECS-SELEX

A primary concern that was explored in parallel with the incorporation of technological advances was the way in which the target was presented to the library. Until then, SELEX procedures were completed using highly purified targets, but selection for cell surface proteins was restricted due to difficulties in the purification of membrane-embedded peptides. Target expressed on cell surface-SELEX (TECS-SELEX) was designed by Ohuchi *et al.* in 2006 for the isolation of aptamers recognizing the TGF-β type III receptor (TbRIII) ectopically expressed on the surface of CHO cells [25]. These modified cells displayed a recombinant form of TbRIII which were utilized as the direct target for a population of RNA oligonucleotides. After incubation, weak- or non-interacting sequences were washed out and remaining aptamers were amplified by RT-PCR. At the end of the process, one of the aptamers had a dissociation constant near 1 nM and competed with TGF-β to bind to the cell surface receptor *in vitro* [25]. Thereby, this change obviates the target purification step, simplifying the overall selection. Three years later, a similar report was made involving the expression of a viral target on the surface of a human cell line [26] instead of expressing a human protein on the surface of CHO cells like described above.

### 6.4. Non-SELEX

In 2006, a technique was developed by Berezovski *et al.* [27] under the principle of Non-Equilibrium Capillary Electrophoresis of Equilibrium Mixtures (NECEEM), a partition method that they previously reported on 2002 as a strategy for the measurement of binding constants for the formation of DNA-Protein complexes. For NECEEM, nucleic acids and protein were combined until equilibrium is reached and the pool was subjected to capillary electrophoresis on a non-equilibrated column leading to a very efficient separation of complexes. NECEEM was successfully coupled to SELEX facilitating the partition step when a recollection window was properly found (CE-SELEX). Non-SELEX is a variation derived from the CE-SELEX in which amplification step is skipped. In the first description, hRas was used as the target for DNA aptamers where an initial PCR reaction was made only to determinate the bulk constant for the naïve library as well as the recollecting window. Afterwards, no amplifications were performed. Then, the recovered hRas-DNA complexes after partition steps were directly incubated with a fresh aliquot of hRas at the same concentration. The authors found that three steps of partitioning in the non-SELEX approach were sufficient to improve the affinity of a DNA library by more than 4 orders of magnitude [27] at the end of the procedure. Affinity values were also higher when compared with those from the enriched library obtained in three rounds of complete CE-SELEX. The time required for the Non-SELEX was just one hour resulting in a fast and economical method for isolation of highly enriched aptamer populations, suggesting that aptamers may be more abundant than they are thought to be [27].

### 6.5. NanoSelection^®^ (nM-AFM SELEX)

Another technological incorporation that improved SELEX procedure was the microscopy. Particularly, atomic force and fluorescence microscopy were combined with small copy number PCR by Peng *et al.* in 2007 [28] to create a new method registered under the name of NanoSelection^®^ (or nano-Manipulator/Atomic Force Microscopy selection, nM-AFM) [4,28]. In this procedure, the authors used a 1:1 mixture of an aptamer against thrombin previously isolated [29] and a nonsense oligonucleotide as a binary library. Both oligos were fluorescently labeled, attached to soft beads and reacted against target-coated substrate. If any of the sequences interacted with thrombin, a strong signal was observed from underneath with an inverted fluorescence microscope meanwhile bead-aptamer-target complexes were detected by the tapping movement of the AFM tip on the upper face of the coated-substrate. The results of this technology were overlaid images where the most brilliant and higher spots corresponded to aptamer-target complexes. These spots were picked-up using a stronger mode of the tip to dragged-out the aptamer from the complex. Finally, individual aptamers were amplified and characterized. The principal advantage of this technique is the possibility to isolate individual aptamers in a single selection cycle from a small pool of random-sequence oligonucleotides [28].

### 6.6. MonoLEX

Just like microscopy and electrophoresis were applied, chromatography also played a role in the development of new approaches to SELEX. Nitsche *et al.* [30] separated aptamer-target complexes using affinity chromatography followed by physical fragmentation of the resin column. The nucleic acids from each section of the column was then amplified and directly characterized avoiding the re-selection step of traditional SELEX leading to a selection in one step, hence it was named MonoLEX [30]. For this particular case, complete Vaccinia virus particles were used as model resulting in a 64-nucleotide DNA aptamer that also recognized other orthopoxviruses family members. Besides, isolated aptamer was capable of inhibiting *in vitro* infection in a concentration-dependent manner [30]. MonoLEX method improves the selection of high affinity aptamers by diminishing the competition between sequences with different affinities during the PCR step, which represents an advantage for the selection [30].

### 6.7. CS-SELEX

The Cell specific SELEX or CS-SELEX is a particular derivation originally developed on 1999 by Homann and Göringer [31] using *Trypanosome brucei* and retaken in 2008 by Shangguan *et al.* [32] who incorporated mass spectrometry. In contrast to TECS-SELEX, neither one of them utilize a system of ectopic expression for target proteins on the cell surface [25]. Homann and Göringer used classic SELEX to isolate RNA aptamers that recognized the invariant surface glycoproteins (IGSs) of the whole living parasite *T. brucei*. These aptamers were able to discriminate between life cycle stages (bloodstream and insect) but unable to distinguish between different strains of parasites. Therefore, their results indicated that the RNA ligands could be used against a greater range of trypanosome strains but they do not specify the protein with which the aptamers interact. In the approach of Shanguann *et al.*, entire cells from established lines were used as targets to determine specific biomarkers when compared to a different cell line. Using CS-SELEX they selected aptamers against cancer-specific cell markers in T-cell acute lymphoblastic leukemia cell line (CCRF-CEM). Briefly, an ssDNA library of 10^15^ sequences was incubated with CCRF-CEM; bound aptamers were collected and immediately passed through counter selection step using control cell lines (e.g., Ramos, a B-cell lymphoma cell line). After 20 cycles of amplification, the aptamer scg8 was isolated and target peptide was analyzed by LC-MS/MS. The resulting peptide sequence was identified as PKT7 when was searched on MASCOT database [32]. Specificity and affinity of scg8 were measured by flow cytometry, indeed adding a new tool that can be incorporated into SELEX. The identification of potential target in a short time made the difference with the original report by CS-SELEX. Aptamers generated by CS-SELEX can serve as specific probes with high affinity for the identified biomarker. This opens an opportunity for diagnostic applications as well as for identification of specific cellular functions [32].

### 6.8. Next Generation SELEX

In 2009 a type of SELEX emerged that is derived from previously reported cDNA/Genomic-SELEX, in this case involving microarrays technology as a detection system of aptamer-target interaction. Reid *et al.* [33] searched for splicing sites into a set of pre-mRNAs by using a library design that displace a 30-nucleotide window in length by increments of 10-nt. The generated oligonucleotides were synthesized as a custom microarray and released from the slide to allowed amplification through universal primer binding sites. Afterwards, the sequences were transcribed to produce small replicas from the selected pre-mRNAs, recognizable by U1-snRNP/PTB, that were subjected to SELEX [33]. Finally, enrichment was then measured as the ratio of oligonucleotide in the bound fraction *versus* that in the starting library. The two-color microarray analysis showed that U1-snRNP joined 5′ splicing site with specificity comparable to the splice donor motif. Selection for PTB resulted in an enrichment of the polypyrimidine tract on the final library. This SELEX application could serve as a tool to explore donor and acceptor splicing sites on pre-mRNAs with clinical relevance such as Duchenne Muscular Dystrophy [34].

### 6.9. Microfluidics SELEX (M-SELEX)

The first concept at microfluidic separation understood as the manipulation of continuous liquid flow through micro-fabricated channels dates back to 2004 when CE-SELEX [23] emerged. In this case, a little sample of target-DNA mixture (up to 5 nL) was injected into a capillary and separated with a high voltage setting the bases of microfluidics SELEX. However, in 2010, microfluidics chips are defined as a system that integrate many functions such as sample preparation, reaction, separation and detection on a single surface (chip) fabricated with minuscule channels where reagents can interact to perform defined reactions. These chips range from millimeters to a few square centimeters in size [35]. Examples of this technology were reported this year by two independent groups, Cho *et al.* used a micro-magnetic device (MMS) and high throughput sequencing to isolate ssDNA aptamers to PDGF-BB (platelet derived growth factor BB) [36]. This method allows the discrimination of aptamers surged as high-affinity target ligands rather than a product of experimental biases. Interestingly, the resulting aptamers had a Kd in the low nM range (<3 nM) obtained with only three rounds of selection and having a ~3–8-fold higher affinity and a ~2–4-fold higher specificity than the aptamers isolated by traditional methods [36]. On the other hand, Huang *et al.* designed and constructed a miniature and automatic platform for SELEX [37]. In their microfluidic system three major modules was integrated: control, magnetic bead-based ssDNA extraction and amplification modules. Each one was used to perform a single step of SELEX (sample incubation and transportation, aptamer screening and fast amplification, respectively) [37]. After interaction of the target protein (C-reactive protein, CRP) with the ssDNA randomized pool in the first module, complexes were separated in the screening module and an on-chip PCR was performed to amplify specific sequences in the last component of the chip. A specific aptamer for CRP with the highest affinity was isolated after five cycles of automatic selection with this device. This microsystem proved to be a fast screening powerful tool that can be used to select aptamers for any target with potential application in clinic.

### 6.10. Multiple-Target High-Throughput SELEX

In 2011, until 31 August, most of reported SELEX assays have been done by applying state-of-the-art technology to multiplex selection and to obtain shortened aptamers as to be quickly and directly applied *in vivo*. Some examples are HAPIscreen [38], Emulsion PCR [39], primer-free SELEX [40] or FACMCE [41]. Basically, the goal is now to obtain several aptamers for different targets reducing times of selection and characterization by combining techniques such as massively parallel sequencing and microfluidics (Multiplexed SELEX) [42]. With the emerge of nanotechnology, it was also possible to miniaturized work areas into small devices (microchips) avoiding cross-contamination due to management of large samples making aptamers relatively easy produced at low cost.

## 7. Bioinformatics Approaches for SELEX

*In silico* analysis has also been used to study the behavior of nucleic acid populations during SELEX, this way, in the last years some variants had been developed such as Neutral-SELEX [43] or DiStRO (a Diversity Standard of Random Oligonucleotides) [44] that permitted to analyze one of the steps of SELEX. This technological addition to the story line of *in vitro* selection led to the description of phenomena not previously known such as biases for a particular nucleotide on the amplification step, progressive changes on melting temperatures that eventually were used to demonstrate evolution of selected pools, and alterations on libraries sequences due to the iterative process itself, even when molecular populations had not been exposed to a particular target. Nevertheless, the details of these adaptations are out of the scope of this review.

## 8. Final Remarks

Over two decades, SELEX has proved to be a convenient method for the isolation of aptamers useful for a wide range of biotechnological and clinical applications. In a typical SELEX experiment, there are as many potential aptamers as combinations of the random positions introduced in a library design. However, after selection, there is a plethora of sequences that are less than those on the initial library but often no SELEX results in a unique product. Usually SELEX renders defined structural families, independently of the selection method employed, and there are groups of ligands with a spectrum of affinities for the same target. The variations of the method mentioned above had helped to avoid the isolation of weak-interacting ligands and now the choice of a particular SELEX depends mainly on how the resulting aptamer is planned to be used. In general, it seems that the technology of *in vitro* selection has reached a point of maximum improvement and the next challenge will be the application of aptamers as molecular tools. Some non-coding RNAs and DNA sequences obtained this way are now being applied in therapeutics and diagnostics fields but there is still a long way to go until aptamers became as widespread as antibodies, although now we know is plausible. This review presented a compilation of some of the modification of SELEX process, but does not include all of the varieties that might be reported in the period of time referred to (1990–2011).

## Figures and Tables

**Table 1:** Timeline of emerging modifications of SELEX.

Year	SELEX type	References *
1990–1993	Classic, Negative	1,2,5
1994	Counter or Subtractive	6,7
1995	Blended (Covalent), Photoselex (crosslinked), cDNA-SELEX	8–10
1996	Spiegelmer isolation	12
1997	*In vivo*	13
1998	Chimeric	14
1999	Multistage, Cell Specific SELEX(CS-SELEX)	15
2000	Beacon aptamers, Indirect	16–18
2001	Toggle	19,20
2002	Expression cassette	21
2003	Tailored-SELEX	22
2004	CE-SELEX	23
2005	FluMAG	24
2006	TECS-SELEX, NON-SELEX (NCEEM)	25,27
2007	NanoSelection^®^ (nM-AFM SELEX), MonoLEX	28,30
2008	CS-SELEX	31,32
2009	Next-generation SELEX	33
2010	Microfluidic-SELEX, Bioinformatics analyses	36,37,43,44
2011	Multiple-target high-throughput SELEX	38–41

* references correspond to the first report of each type of SELEX;

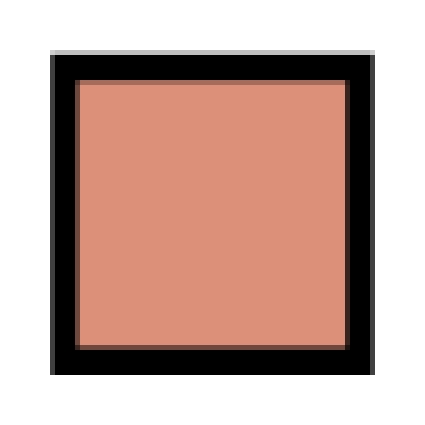
 Setting the ground;

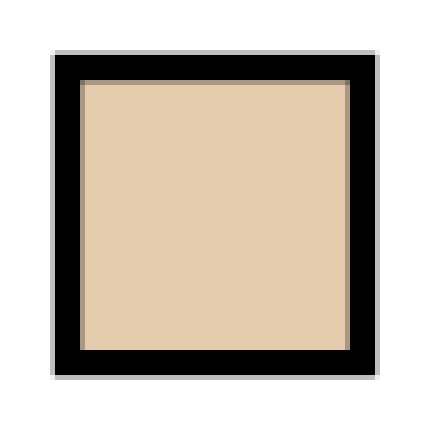
 Improving the libraries;

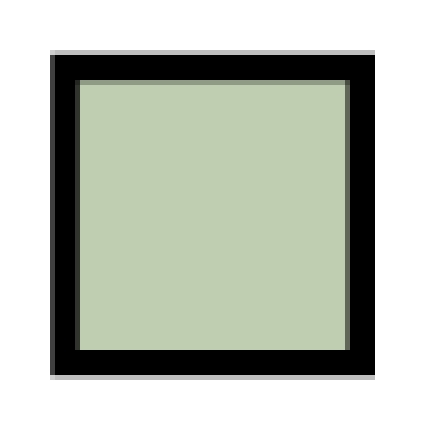
 Entering the cell environment;

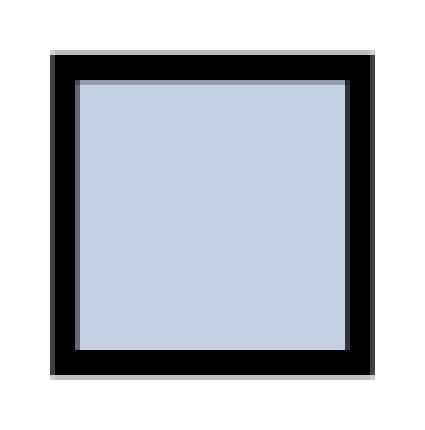
 Regulation and detection;

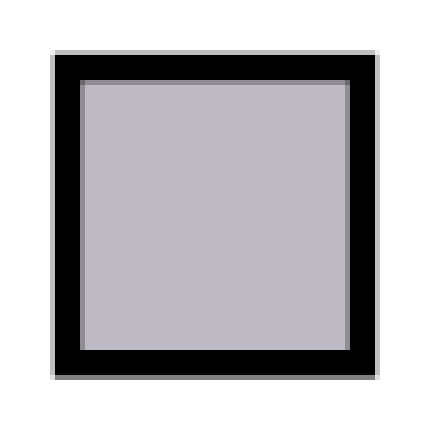
 Updating SELEX with modern Technologies.
